# Surgical Repair of Lower Extremity Vascular Injuries in Children: Two Cases

**DOI:** 10.1155/2014/606574

**Published:** 2014-08-25

**Authors:** Mehmet Tasar, Nur Dikmen Yaman, Cahit Saricaoglu, Zeynep Eyileten, Bulent Kaya, Adnan Uysalel

**Affiliations:** Department of Cardiovascular Surgery, Heart Center, Ankara University School of Medicine, Dikimevi, 06340 Ankara, Turkey

## Abstract

The use of percutaneous devices is commonplace for the treatment of many congenital heart defects. However, there are some situations where procedure-related complications are encountered and surgical help is required to ameliorate this issue. Vascular injury due to transcatheter intervention is a significant and challenging complication and occasionally requires surgical approach. In this case report, we aimed to present successful surgical management of vascular injuries associated with percutaneous interventions in children.

## 1. Introduction

Lower extremity arterial injuries that require surgical repair are not common in children. They are mostly seen due to iatrogenic trauma during diagnostic or therapeutic femoral artery catheterization [1]. Vascular surgery is the most important treatment modality for this complication. In this study, we aimed to present our surgical approaches and also short- and long-term results of surgical repair of vascular injuries in two children.

## 2. Case  1

**Figure 1:**
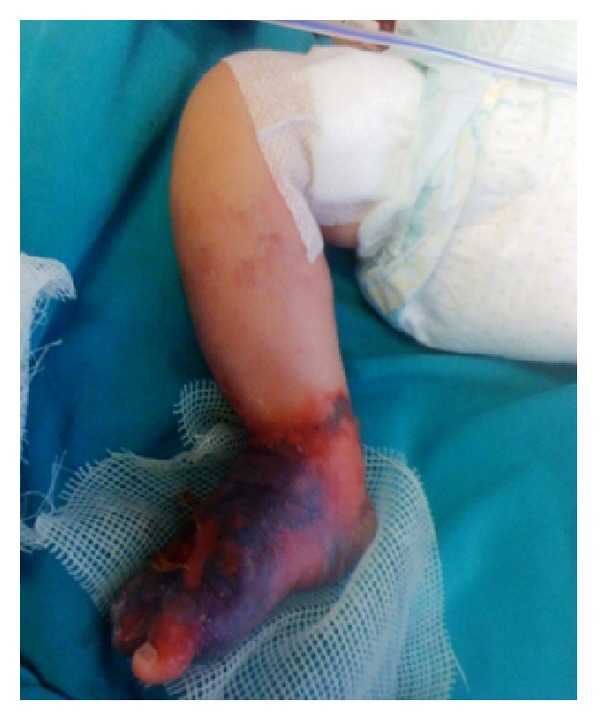
Foot ischemia before vascular surgery.

A 10-day neonate was admitted to Pediatric Cardiology Clinic with patent ductus arteriosus (PDA). PDA was about 1.5 mm and coil embolization was planned. In catheterization room, this procedure was complicated by a misplacement of coil towards the left pulmonary artery. After percutaneous interventions to the right leg, several attempts were made to remove coil but it was not achieved and the patient was directed to cardiovascular surgery. Following median sternotomy, the coil material was removed via pulmonary arteriotomy under cardiopulmonary bypass and then PDA was ligated. In intensive care unit, right foot ischemia was observed. Because of its progressive expansion towards the ankle (Figure 1), surgical intervention was planned.

**Figure 2:**
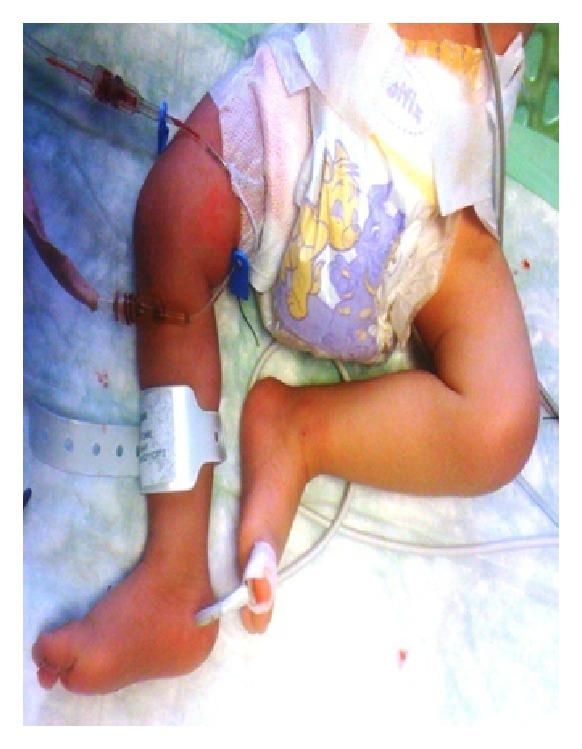
No ischemia after vascular surgery.

Femoral artery was found and dissected under general anesthesia. The vessel was damaged secondary to percutaneous interventions. Embolectomy was tried proximally and distally with 2 Fr Fogarty catheter, thrombus was removed, and patent flow of femoral artery was detected. The damaged portion of femoral artery was resected and interposed by a great saphenous vein graft which was harvested from ipsilateral side. Two days later, foot ischemia was regressed (Figure 2). In 15-day period, total reperfusion was established and distal peripheral pulses were palpable. Colored Doppler ultrasonography (USG) showed that flow of femoral artery was sufficient. The patient was discharged with low molecular weight heparin. During his long-term followup, colored Doppler USG showed that graft was patent, and no ischemia was observed.

## 3. Case  2

A two-year old girl with patent ductus arteriosus and pulmonary valvular stenosis was hospitalized in Pediatric Cardiology Clinic for transcatheter intervention. She was taken to intervention with general anesthesia; 5F pigtail catheter was introduced from right femoral artery to arcus aorta. Pulmonary valvuloplasty was performed with 12 mm × 3 cm sized balloon valvuloplasty catheter. After arcus aorta injection, 2.2 mm Krichenko Type A PDA was viewed. 3 × 6 mm Amplatzer duct occluder was placed by transarterial route. After intervention, sudden desaturation and bradycardia were detected and transthoracic echocardiography showed that the occluder was displaced into pulmonary artery. Device was caught by 10 mm sized Gooseneck snare and brought into the femoral artery. She was transferred to our clinic for open surgery.

In general anesthesia, following midline laparotomy incision, Amplatzer occluder was found in iliac artery and removed. Because of vessel wall injury due to several attempts for percutaneous retrieval of device, arterial flow was not enough so patient underwent right iliofemoral bypass grafting with 8 mm polytetrafluoroethylene (PTFE) graft. Two years later, duplex-ultrasonography showed that bypass graft was patent and also physical examination was normal.

## 4. Discussion

Arterial reconstruction of the lower extremities in children and neonates is significantly unusual. The knowledge in literature is mostly produced from adult lower extremity revascularization with vein grafts. Moreover, most pediatric lower extremity vascular pathologies are due to iatrogenic trauma [1]. Direct arterial interventions may cause injuries of associated vessel walls [2]. Our cases are the examples of iatrogenic vascular injuries due to transcatheter interventions.

Percutaneous closure of secundum atrial septal defect and patent ductus arteriosus by various devices has been approved as an effective and safe treatment procedure for congenital heart diseases. Somehow, potential early and late complications like device embolization may occur [3]. Close contact with the cardiovascular surgeons makes sure that urgent open heart and vascular surgery is readily available if the percutaneous rescue maneuver fails or results in unwanted complications [4]. Decisions determining the operative technique of pediatric vascular injuries must consider vessel size and future growth potential [5].

Many methods are available to reconstruct arteries including primary repair, vein patch angioplasty, and interposition repair using reversed greater saphenous vein (GSV), other autologous vein, or synthetic grafts (PTFE or Dacron) [6]. So we used two different techniques, autologous and PTFE synthetic vein graft, and long-term results were similar. There are reservations about using saphenous vein for arterial repair in the pediatric population. Saphenous vein is a good choice but late aneurysmal degeneration occasionally occurs in children [1].

A few information is known about the outcomes of pediatric vascular reconstructions because of the small number of studies in this subject. More prospective clinical trials are needed.

As the conclusion of this report, vascular surgery can be required after percutaneous interventions in children. Cooperation between pediatric cardiologists and pediatric cardiovascular surgeons should be kept in mind to dodge the least injury. During the intervention or catheterization, operation room should be ready.
